# Artificial Intelligence in Atrial Fibrillation: From Early Detection to Precision Therapy

**DOI:** 10.3390/jcm14082627

**Published:** 2025-04-11

**Authors:** Paschalis Karakasis, Panagiotis Theofilis, Marios Sagris, Konstantinos Pamporis, Panagiotis Stachteas, Georgios Sidiropoulos, Panayotis K. Vlachakis, Dimitrios Patoulias, Antonios P. Antoniadis, Nikolaos Fragakis

**Affiliations:** 1 Second Department of Cardiology, Hippokration General Hospital, Aristotle University of Thessaloniki, 54642 Thessaloniki, Greece; staxteasp@gmail.com (P.S.); aantoniadis@gmail.com (A.P.A.); fragakis.nikos@googlemail.com (N.F.); 2 First Cardiology Department, School of Medicine, Hippokration General Hospital, National and Kapodistrian University of Athens, 11527 Athens, Greece; panos.theofilis@hotmail.com (P.T.); masagris1919@gmail.com (M.S.); konstantinospab@gmail.com (K.P.); vlachakispanag@gmail.com (P.K.V.); 3 Department of Cardiology, Georgios Papanikolaou General Hospital, Leoforos Papanikolaou, 57010 Thessaloniki, Greece; sidiropoulos.georges@gmail.com; 4 Second Propedeutic Department of Internal Medicine, Faculty of Medicine, School of Health Sciences Aristotle, University of Thessaloniki, 54642 Thessaloniki, Greece; dipatoulias@gmail.com

**Keywords:** atrial fibrillation, artificial intelligence, catheter ablation, electroanatomic mapping, machine learning, electrogram analysis, predictive modeling

## Abstract

Atrial fibrillation (AF) is the most prevalent cardiac arrhythmia, associated with significant morbidity, mortality, and healthcare burden. Despite advances in AF management, challenges persist in early detection, risk stratification, and treatment optimization, necessitating innovative solutions. Artificial intelligence (AI) has emerged as a transformative tool in AF care, leveraging machine learning and deep learning algorithms to enhance diagnostic accuracy, improve risk prediction, and guide therapeutic interventions. AI-powered electrocardiographic screening has demonstrated the ability to detect asymptomatic AF, while wearable photoplethysmography-based technologies have expanded real-time rhythm monitoring beyond clinical settings. AI-driven predictive models integrate electronic health records and multimodal physiological data to refine AF risk stratification, stroke prediction, and anticoagulation decision making. In the realm of treatment, AI is revolutionizing individualized therapy and optimizing anticoagulation management and catheter ablation strategies. Notably, AI-enhanced electroanatomic mapping and real-time procedural guidance hold promise for improving ablation success rates and reducing AF recurrence. Despite these advancements, the clinical integration of AI in AF management remains an evolving field. Future research should focus on large-scale validation, model interpretability, and regulatory frameworks to ensure widespread adoption. This review explores the current and emerging applications of AI in AF, highlighting its potential to enhance precision medicine and patient outcomes.

## 1. Introduction

Atrial fibrillation (AF) is the most common cardiac arrhythmia and a significant contributor to global morbidity and mortality [1,2,3,4,5]. Its incidence is expected to increase substantially in the coming years, placing a considerable economic and operational strain on healthcare systems [6]. Despite advancements in AF management, persistent challenges in early detection, risk stratification, and treatment optimization highlight the need for innovative approaches to enhance clinical outcomes.

Artificial intelligence (AI) is increasingly being integrated into transformative technologies across multiple domains, including healthcare [7]. Its capacity to process and analyze large-scale medical datasets with computational efficiency and precision has enabled applications that often exceed human performance, particularly in fields reliant on complex pattern recognition, such as diagnostic radiology [7]. Despite these advancements, the direct application of AI-driven software in therapeutic decision making and procedural guidance remains relatively unexplored, representing a largely untapped area of potential innovation [8,9].

**Figure 1:**
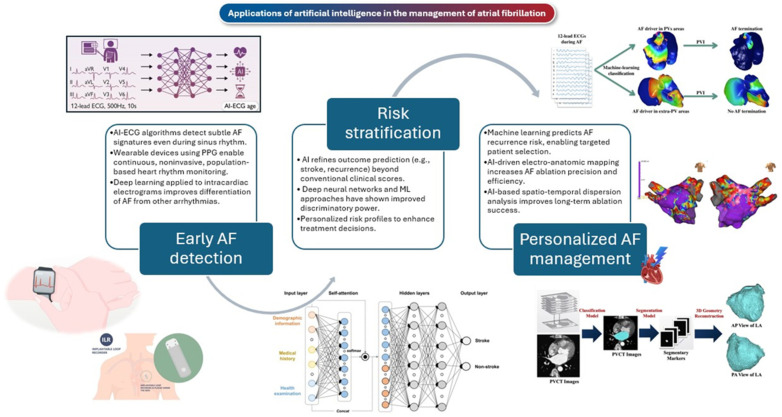
The role of artificial intelligence (AI) in atrial fibrillation (AF) management is expanding, with advancements in risk stratification, personalized medical therapy, and catheter ablation strategies. AI-driven electrocardiographic and imaging models have shown promise in improving stroke risk prediction compared to conventional clinical scores. Machine learning (ML) algorithms contribute to refining neurological deterioration risk assessment in AF-related stroke and assist in predicting cardioversion success. In personalized AF management, AI is being applied to optimize anticoagulation and antiarrhythmic therapy by improving drug dosing, supporting medication adherence, and enabling real-time monitoring. In catheter ablation, AI-powered electroanatomic mapping, recurrence risk prediction, and real-time procedural guidance are being explored to enhance procedural precision and outcomes. While these advancements are promising, challenges remain in integrating AI into clinical practice. Further validation, regulatory considerations, and real-world implementation studies are needed to support broader adoption. AI has the potential to improve AF diagnosis, risk assessment, and treatment decisions, contributing to a more data-driven and individualized approach to AF management.

AI and machine learning have emerged as transformative tools in modern cardiology, offering unprecedented capabilities in diagnosing and managing AF (Figure 1) [10]. AI-powered electrocardiographic screening has demonstrated the ability to identify AF even in asymptomatic individuals, who constitute approximately one-third of the AF population [11] and exhibit a prognosis comparable to symptomatic cases [12], thereby enabling earlier intervention and potentially improving clinical outcomes [13]. Additionally, AI-driven wearable technologies and photoplethysmography-based detection systems have enabled continuous, real-world rhythm monitoring, expanding AF screening beyond clinical settings [14]. Beyond detection, AI-based predictive models have been developed to refine risk stratification by integrating clinical characteristics, electronic health records, and multimodal physiological data [15]. These models have demonstrated potential in predicting AF onset, stroke risk, and treatment response, offering a data-driven approach to improving decision making for anticoagulation and rhythm management. Additionally, AI is increasingly contributing to individualized AF therapy, optimizing anticoagulation strategies and catheter ablation procedures [16]. Recent advancements in AI-driven electroanatomic mapping and real-time procedural guidance hold promise for enhancing ablation success rates and minimizing AF recurrence [17,18,19,20], paving the way for more precise and effective treatment strategies.

A comprehensive literature search was performed across PubMed/MEDLINE, Cochrane, Embase, and Google Scholar, covering publications from the inception of each database through March 2025. Eligible sources included original research articles, clinical trials, meta-analyses, and systematic reviews that examined the application of artificial intelligence in atrial fibrillation, particularly in the areas of early detection, risk stratification, and therapeutic intervention. This review provides a detailed overview of the current and emerging roles of AI in AF management, with an emphasis on its integration into individualized care pathways. By synthesizing the available evidence, the review offers insights into the evolving landscape of AI-driven AF care and its potential to advance precision medicine and improve patient outcomes.

## 2. AI-Powered Electrocardiographic Screening for the Early Detection of AF

AI-enhanced electrocardiography (AI-ECG) has emerged as a novel approach for predicting and identifying AF, even in patients with sinus rhythm [21]. Given the gradual progression and often subtle nature of cardiac structural alterations—along with their corresponding electrocardiographic manifestations—AI-driven algorithms are inherently well-equipped to recognize these patterns. These computational models have the potential to detect abnormalities that may evade both trained and untrained human observers, thereby enhancing early diagnosis and risk stratification [22].

An AI-ECG algorithm was developed using approximately 650,000 ECG recordings from the Mayo Clinic to facilitate the identification of paroxysmal AF in patients maintaining sinus rhythm [21]. A single AI-ECG assessment demonstrated an area under the receiver operating characteristic curve (AUC) of 0.87 for detecting underlying paroxysmal AF, which improved to an AUC of 0.90 when incorporating all ECGs recorded within the first month of each patient’s designated observation period (i.e., the 31 days preceding the first documented AF episode) [21]. In a subsequent investigation, the predictive utility of AI-ECG for future AF onset was evaluated in individuals without a prior AF diagnosis, utilizing data from the Mayo Clinic Study of Aging [23]. The predictive performance of AI-ECG was further compared to the CHARGE-AF risk score [23]. Among patients for whom the AI-ECG model assigned a >50% probability of AF development, the cumulative incidence of AF at 2 and 10 years was 21.5% and 52.2%, respectively, demonstrating a predictive capacity comparable to that of CHARGE-AF [23].

A deep-learning-based AI-ECG risk stratification model demonstrated strong predictive performance for five-year AF-free survival, achieving an AUC of 0.823 across three independent cohorts comprising over 80,000 individuals [24]. Notably, saliency mapping identified the P-wave segment as the algorithm’s primary region of interest, a biologically plausible finding that aligns with established electrophysiological principles [24]. Building on this, Wu et al. leveraged the PhysioNet database, which includes 30 min ECG recordings from 100 patients (50 with underlying paroxysmal AF) in sinus rhythm, to develop and compare AI models using three distinct machine learning approaches [25]. Among the evaluated techniques—bagging, AdaBoost, and stacking—the stacking model exhibited the highest predictive performance, achieving an AUC of 0.911 (range across models: 0.88–0.91). However, this model has yet to undergo external validation [25].

Beyond predictive modeling, AI-ECG has been explored in the context of embolic stroke of undetermined source (ESUS), a condition in which silent, underlying AF is often suspected as the etiological culprit [26]. Although AI-ECG-derived AF probability did not significantly differ between patients with ESUS and those with strokes attributed to alternative mechanisms, individuals with ESUS who exhibited a high AI-ECG-predicted probability of AF demonstrated a markedly increased likelihood of subsequent AF detection during ambulatory monitoring [26].

Of note, the clinical utility of AI-ECG for AF detection was recently validated in a prospective interventional trial, demonstrating its potential in guiding targeted screening strategies [13]. Investigators integrated AI-ECG-derived probability scores for paroxysmal AF with electronic health record (EHR)-based patient characteristics to identify individuals most likely to benefit from extended ECG monitoring for AF detection [13]. Eligible participants had no prior AF diagnosis but exhibited both an elevated AI-ECG-predicted probability of AF during sinus rhythm and clinical risk factors suggesting a need for anticoagulation if AF were detected (e.g., elevated CHA_2_DS_2_-VASc score). Notably, clinical risk assessment incorporated both structured and unstructured EHR data, leveraging natural language processing to extract relevant information [27]. Among patients stratified as high-risk based on AI-ECG predictions, the likelihood of AF diagnosis on 30-day ambulatory monitoring was fivefold higher than in those classified as low-risk by AI-ECG probability [13]. These findings underscore the potential of AI-ECG to enhance AF screening strategies through individualized risk stratification and data-driven patient selection.

## 3. AI in Wearable Photoplethysmography for Real-Time AF Detection

The ECG remains the gold standard for AF diagnosis; however, the increasing prevalence of consumer-grade wearable devices has introduced a paradigm shift in AF detection [28,29]. Photoplethysmography (PPG) technology enables real-time, automated rhythm assessment in non-clinical settings, utilizing wearable devices and smartphones to facilitate continuous cardiac monitoring [30,31]. The detection algorithms employed by commercially available PPG-based technologies (e.g., Apple, Fitbit, Huawei Watch) leverage a combination of temporal and morphological signal characteristics to differentiate AF from sinus rhythm, providing users with irregular rhythm notifications [32].

The Apple Heart Study exemplified the feasibility of decentralized AF screening through smartwatch-based remote monitoring [33]. In this large-scale study, participants downloaded a dedicated application that continuously assessed heart rhythm irregularities via PPG. Individuals who received notifications of an irregular pulse were subsequently considered for confirmatory remote monitoring to establish an AF diagnosis. Among those who underwent further testing, 34% were diagnosed with AF, and 84% of irregular pulse notifications were concordant with AF on ECG confirmation [33].

Similarly, the Huawei Heart Study provided additional validation of wearable PPG-based AF detection in a decentralized cohort [34]. Among participants notified of “suspected AF”, 87% (*n* = 227) were ultimately confirmed to have AF, with a positive predictive value approaching 92% [34]. These landmark studies highlight the scalability of PPG-integrated consumer technologies in AF screening, offering an efficient pathway for clinical data acquisition beyond traditional healthcare settings.

In a prospective, real-world study, Mannhart et al. [35] validated the diagnostic accuracy of five commercially available wearable smart devices—Apple Watch 6, Samsung Galaxy Watch 3, Withings ScanWatch, Fitbit Sense, and AliveCor KardiaMobile—for AF detection, using a physician-interpreted 12-lead ECG as the gold standard. Among 201 enrolled patients, AF was present in 31%. Sensitivity and specificity varied across devices, with the Apple Watch 6 and Samsung Galaxy Watch 3 demonstrating the highest sensitivity (85%), while the Withings ScanWatch exhibited the lowest sensitivity (58%) [35]. Notably, inconclusive tracings, which required manual physician review, occurred in up to 26% of cases, significantly impacting overall diagnostic accuracy [35]. Despite these limitations, manual interpretation of smart device tracings yielded a diagnostic accuracy of 98–100%, underscoring the potential of these technologies in AF screening when combined with expert review [35].

The computational methodologies underlying PPG-based AF detection have evolved from traditional regression-based models with manual feature extraction to more sophisticated machine learning frameworks, including support vector machines, decision trees, and deep learning architectures [36,37]. While deep learning approaches enable autonomous feature extraction and improved classification accuracy, they require substantial datasets for training and validation. Notably, the proprietary nature of algorithms utilized in commercially available platforms, such as those in the Apple Heart Study and Huawei Heart Study, precludes external validation and limits transparency regarding their exact methodological frameworks [34,38].

Despite the promising potential of wearable-based AF detection, several critical considerations must be addressed before widespread clinical integration. One significant limitation is the susceptibility of PPG signals to motion artifacts and ambient noise, which can contribute to false-positive detections [39]. Additionally, PPG-based algorithms may lack the specificity to differentiate AF from other atrial arrhythmias, such as atrial flutter or multifocal atrial tachycardia, raising concerns regarding diagnostic accuracy [39]. Moreover, the adoption of these technologies has been predominantly driven by younger, tech-savvy populations, whereas older individuals—who face a heightened arrhythmogenic and thromboembolic risk—may be underrepresented in validation studies. Recognizing this gap, the eBRAVE-AF trial specifically evaluated smartphone-based PPG wave analysis in an older cohort (aged 50–90 years), comparing its performance against conventional symptom-based screening [30]. Notably, digital app-based screening significantly enhanced the detection of clinically relevant AF, with an odds ratio of 2.1 in Phase I and 2.8 in Phase II, underscoring its potential utility in high-risk populations [40].

As PPG-based technologies continue to evolve through iterative refinements and rigorous validation in clinically relevant cohorts, their diagnostic performance is expected to improve. These advancements may facilitate broader acceptance and large-scale AF screening via consumer-driven wearable solutions, ultimately redefining the landscape of arrhythmia surveillance by bridging the gap between traditional clinical diagnostics and real-world, continuous health monitoring.

## 4. AI-Based Detection of AF in Patients with Implantable Devices

Implantable cardiac devices frequently encounter challenges in accurately differentiating AF from other atrial arrhythmias, such as atrial flutter, atrial tachycardia, or premature atrial ectopic beats, often leading to misclassification based on rate and signal irregularity in intracardiac electrograms (EGMs). To address this limitation, Rodrigo et al. developed a deep learning algorithm designed to enhance the precision of AF identification by leveraging advanced EGM feature analysis [41]. The deep learning model demonstrated superior discriminatory performance, achieving an AUC of 0.95–0.97, depending on whether unipolar or bipolar EGM data were utilized. This markedly outperformed traditional classification methods relying on single EGM features, which exhibited AUCs ranging from 0.67 to 0.75 [41]. These findings underscore the potential of deep learning as a powerful analytical tool for improving AF detection in cardiac implantable electronic devices, warranting further investigation into its clinical integration to refine arrhythmia classification and reduce diagnostic errors.

## 5. AI-Based Prediction of AF Utilizing Clinical Characteristics

Patient-specific clinical characteristics, including age, sex, and underlying comorbidities, serve as established predictors of AF risk. Multivariable risk stratification models, such as the CHARGE-AF score, have been extensively validated and are widely employed to estimate the likelihood of AF development [42,43,44]. While AI and ML methodologies offer the potential to enhance these predictive models by leveraging electronic health record (EHR) data, the degree of improvement in predictive performance remains variable [45].

A large-scale investigation conducted at the University of Colorado utilized an ML model to analyze over 200 EHR-derived clinical features associated with AF risk in a cohort of two million patients [46]. The resultant predictive algorithm demonstrated an AUC of 0.79 for AF detection over a six-month follow-up period. However, this predictive accuracy was comparable to that of conventional, non-AI-based clinical AF risk scores, which exhibited AUCs ranging from 0.71 to 0.78 [46,47,48,49]. In contrast, two large-scale studies conducted in the United Kingdom employed similar ML techniques yet achieved superior predictive performance relative to the CHARGE-AF score, with AUCs of 0.827 and 0.725, respectively [50,51]. The authors hypothesized that the improved model performance was attributable to the utilization of higher-quality datasets with extended follow-up durations, as well as the integration of advanced analytical methodologies, such as modeling time-dependent covariates [50,51,52].

Beyond AF prediction, there is growing interest in leveraging AI-based models to characterize the longitudinal trajectory of AF progression and its clinical course [53,54]. The integration of AI-enhanced EHR-based prognostic models into clinical workflows may ultimately refine screening strategies and optimize individualized treatment approaches for AF management.

## 6. AI for Risk Stratification in Atrial Fibrillation

In the context of newly diagnosed AF, AI and machine learning methodologies hold promise for refining risk stratification, particularly in predicting clinical outcomes such as ischemic stroke and the likelihood of successful cardioversion.

Jung et al. conducted a large-scale analysis utilizing data from 754,949 patients with paroxysmal AF sourced from the Korean National Health Insurance Service database to identify clinical determinants of ischemic stroke risk [15]. Using logistic regression, they identified 48 features associated with stroke occurrence and subsequently developed a deep neural network (DNN) model to enhance predictive accuracy. Upon validation, the DNN model demonstrated superior discriminatory performance compared to the CHA_2_DS_2_-VASc score, as evidenced by a higher area under the receiver operating characteristic curve (AUROC) value (0.727 ± 0.003 vs. 0.651 ± 0.007), underscoring its potential in refined stroke risk stratification [15].

In a complementary approach, Li et al. explored the microvascular and hypoxic characteristics implicated in ischemic stroke pathogenesis among AF patients by leveraging retinal imaging at specific wavelengths (548 nm, 605 nm, and 810 nm) [55]. A DNN model was trained to assess stroke risk based on these spectral retinal images, demonstrating a predictive accuracy exceeding 78%. Notably, the model trained with 605 nm spectral images exhibited greater stability in ischemic stroke detection, with the multispectral composite model achieving an AUROC of 0.954, surpassing the performance of single-spectral models [55].

Despite significant advancements in acute stroke management, early neurological deterioration (END) remains a major clinical challenge, characterized by considerable heterogeneity and complexity [56,57]. In an effort to improve risk prediction, Kim et al. developed an ML-based model for END in stroke-associated AF, comparing various algorithms, including support vector machines and light gradient boosting machines (LightGBM) [58]. Among these, LightGBM demonstrated the highest predictive performance, achieving an AUROC of 0.772, highlighting the potential of ML-based approaches in refining prognostic assessment and guiding early intervention strategies [58].

Similarly, AI and ML approaches have been explored to identify clinical determinants of cardioversion success. Vinter et al. evaluated a sex-specific predictive model integrating ML and logistic regression to assess the likelihood of successful electrical cardioversion [59]. This model incorporated diverse variables, including comorbidities, echocardiographic parameters, and pharmacological therapies, using a cohort of 332 women and 790 men. However, both ML-based and traditional regression analyses yielded only modest predictive capabilities, with AUC values ranging from 0.56 to 0.60 in both sexes [59].

Further efforts have sought to validate ML-based algorithms for predicting cardioversion outcomes in patients referred for electrical cardioversion (n = 429), comparing AI-driven predictions against established risk scores such as CHA_2_DS_2_-VASc and HATCH, which have previously been implicated in forecasting AF recurrence following cardioversion [60,61,62]. The study findings were mixed. While ML models demonstrated superior predictive performance in estimating six-month AF recurrence, rhythm control, and pharmacological cardioversion success, they were less effective than CHA_2_DS_2_-VASc and HATCH scores in predicting electrical cardioversion success [62]. Despite requiring further external validation and optimization before clinical implementation, this study highlighted the utility of feature importance analysis in identifying key patient-specific clinical attributes that contribute to AI-based predictive models.

## 7. AI for Personalized AF Management

### 7.1. Medical Therapy

The application of AI in AF management extends beyond risk prediction to optimizing pharmacotherapy, particularly in antiarrhythmic and anticoagulant therapy. AI-driven models offer novel strategies for individualized dosing, toxicity monitoring, and medication adherence, addressing critical challenges in AF pharmacological management [63,64,65,66,67].

Dofetilide, a class III antiarrhythmic agent used for rhythm control in AF, necessitates careful therapeutic monitoring due to its proarrhythmic potential [68]. While the QT interval serves as a surrogate marker for plasma drug concentration, it has inherent limitations [69]. To improve predictive accuracy, Attia et al. developed a deep learning algorithm to model the relationship between QTc interval morphology and dofetilide plasma levels [70]. Their model demonstrated a significant correlation between DL-derived QTc modifications and measured plasma concentrations, surpassing conventional QT-based estimation techniques [70].

Anticoagulation therapy remains central to AF management for stroke prevention; however, precise dosing is imperative to mitigate the competing risks of thrombosis and bleeding. AI has shown promise in refining anticoagulation strategies through personalized dosing models. Heemoon Lee et al. [71] developed a deep neural network (DNN)-based model to predict international normalized ratio (INR) fluctuations and generated an individualized warfarin dosage-INR reference table. Their AI-driven dosing algorithm outperformed clinician-based estimates in predicting future INR values, highlighting its potential to enhance warfarin titration precision [71].

Additionally, Cheng Chen et al. employed multiple machine learning techniques, including logistic regression, random forest, and extreme gradient boosting (XGBoost), to identify risk factors for bleeding complications and construct predictive models for anticoagulant-associated hemorrhagic events [72]. Among these approaches, the XGBoost model exhibited the highest predictive accuracy and discriminatory capacity for bleeding risk, demonstrating its potential to support individualized anticoagulation strategies, particularly in elderly patients [72].

Medication non-adherence remains a significant barrier to effective anticoagulation therapy in AF, with only modest improvements observed following the introduction of direct oral anticoagulants [73]. In an innovative approach, Labovitz et al. [16] integrated smartphone-based AI technology to monitor anticoagulant adherence in real time. Their intervention achieved a 100% adherence rate in the AI-monitored cohort, compared to 50% in patients managed through traditional methods, underscoring the role of AI-enhanced monitoring in improving compliance and therapeutic outcomes [16].

### 7.2. Catheter Ablation Therapy

Radiofrequency catheter ablation remains a cornerstone in the management of AF, with procedural success heavily reliant on the precise anatomical delineation of the left atrium (LA). While three-dimensional magnetic resonance imaging (MRI) offers detailed visualization of cardiac structures, the accurate segmentation of the LA remains challenging due to anatomical complexity and image quality variability (Table 1) [74,75].

To address these limitations, Chen et al. [84] developed a deep convolutional neural network (CNN) model trained on cardiac computed tomography (CT) images from 97 patients, enabling automated segmentation and three-dimensional reconstruction of the LA. This AI-driven model achieved an exceptional accuracy of 99.0% in identifying LA structures within CT images, with a sensitivity of 99.3% and a specificity of 98.7% [84]. Similarly, Liu et al. integrated a CNN with a recurrent neural network to enhance segmentation precision, further optimizing procedural planning for AF ablation [85].

Beyond anatomical delineation, personalized ablation strategies are pivotal in improving treatment efficacy. Muffoletto et al. [86] employed a two-dimensional atrial tissue model to simulate conventional AF ablation approaches and trained a deep neural network (DNN) to classify and predict the optimal catheter ablation strategy. The AI-based model achieved an overall accuracy of 79% in identifying the most effective ablation pattern, highlighting the potential of computational modeling to refine patient-specific procedural planning [86].

Despite advancements in catheter ablation, AF recurrence remains a substantial clinical challenge, with recurrence rates reaching up to 75% in patients with persistent AF [87,88,89,90,91]. AI-driven predictive modeling has emerged as a promising approach for identifying patients at high risk of recurrence, facilitating early intervention strategies following catheter ablation [83]. The integration of neural networks into electroanatomic cardiac mapping has demonstrated significant potential in improving the precision of arrhythmia localization and ablation targeting. Convolutional neural networks (CNNs) have been employed to predict phase maps, rotor positions, and phase singularities, achieving high accuracy levels approaching 95% [92]. Notably, these AI-driven models exhibit greater resilience to noise and improved generalizability across different species, outperforming conventional phase mapping techniques in diverse electrophysiological conditions [92]. Similarly, deep-learning-based frameworks have been applied to unipolar electrogram (EGM) signals, automating focal source detection for potential ablation targets with diagnostic performance comparable to that of experienced electrophysiologists [93]. Tang et al. [94] developed a CNN-based multimodal fusion framework incorporating intracardiac electrogram data, electrocardiographic signals, and clinical variables to predict AF recurrence one year post-ablation, achieving an area under the receiver operating characteristic curve of 0.859. Additionally, Liu et al. devised a deep learning model to predict the presence of non-pulmonary vein (NPV) triggers, which are implicated in AF recurrence [83]. Their model demonstrated an accuracy of 82.4%, with a sensitivity of 64.3% and a specificity of 88.4%, allowing for the early identification of high-risk patients and guiding targeted interventions to mitigate recurrence risk following catheter ablation [83].

Recent advancements in AI-driven real-time software have introduced the ability to recognize and classify specific AF electrical patterns, particularly spatio-temporal electrogram dispersion, which may help overcome existing challenges in AF ablation [18,19]. Spatio-temporal dispersion is characterized by clusters of three or more adjacent bipolar electrograms forming localized sequential activation within a distinct atrial region, spanning the entire AF cycle length. This pattern is suggestive of localized reentrant conduction, which may contribute to the initiation or maintenance of AF [95]. Building on this concept, the TAILORED-AF trial [17], a multicenter, randomized controlled study, evaluated the efficacy of an AI-guided tailored ablation strategy compared to conventional pulmonary vein isolation (PVI) only in patients with persistent and long-standing persistent AF. A total of 374 patients were randomized in a 1:1 ratio to undergo either AI-assisted ablation targeting spatio-temporal electrogram dispersion in addition to PVI or a standard PVI-only approach [17]. At 12 months post-procedure, freedom from AF—the primary endpoint—was significantly higher in the AI-tailored ablation group (88%) compared to the PVI-only group (70%, log-rank *p* < 0.0001) [17]. While there was no statistically significant difference in freedom from any atrial arrhythmia after a single procedure in the overall per-protocol population (HR, 0.81; 95% CI, 0.56–1.19), a favorable trend emerged after one or two procedures (HR, 0.62; 95% CI, 0.38–1.01) [17]. Importantly, in the pre-specified subgroup of patients with AF duration ≥ 6 months, the AI-guided approach demonstrated a significant benefit both after a single procedure (HR, 0.60; 95% CI, 0.37–0.97) and after one or two procedures (HR, 0.50; 95% CI, 0.28–0.90) [17]. Procedure times in the AI-guided arm were approximately twice as long as in the PVI-only group; however, safety outcomes remained comparable, with no increase in adverse events [17]. Notably, the TAILORED-AF trial represents a landmark in the field—it is the first international, large-scale randomized controlled trial to demonstrate the superiority of a personalized, AI-guided ablation strategy over conventional PVI in patients with persistent AF.

These findings highlight the emerging role of AI in advancing AF ablation strategies by integrating precise anatomical segmentation, predictive modeling for recurrence, and electroanatomic mapping optimization. Through data-driven methodologies, AI could enhance ablation precision, procedural efficiency, and patient selection, ultimately complementing conventional interventional techniques and contributing to a more personalized and outcome-driven approach in AF management.

## 8. Conclusions

AI is rapidly transforming the landscape of AF management, from early detection and risk stratification to personalized therapy and procedural optimization. AI-driven electrocardiographic screening, wearable technology, and predictive modeling have shown promise in identifying AF earlier, improving risk stratification, and refining treatment strategies. Furthermore, AI-guided approaches in catheter ablation, electroanatomic mapping, and real-time procedural guidance have enhanced precision and efficiency, paving the way for more targeted interventions.

Despite these advancements, several challenges remain before widespread clinical adoption can be achieved. A significant limitation in the current landscape of AI-driven atrial fibrillation detection lies in the proprietary nature of several commercially available algorithms, such as those developed by Apple and Huawei. These systems often operate as “black boxes”, with their internal architectures, feature selection processes, and model training data remaining undisclosed. This lack of transparency hampers independent reproducibility, external validation, and head-to-head comparison, all of which are critical for establishing clinical reliability and generalizability across diverse patient populations. Furthermore, the closed-source nature of these platforms poses challenges for regulatory oversight, as health authorities may not have full access to the algorithmic logic underpinning medical decisions. This raises important questions regarding accountability in the event of diagnostic errors or adverse outcomes. Legal and ethical concerns also emerge when proprietary algorithms are used in direct-to-consumer health applications without clear guidance on liability, data ownership, or informed consent. As AI adoption accelerates in cardiovascular care, addressing these gaps through more transparent, auditable, and regulation-ready models will be essential to ensure safe and equitable clinical implementation.

Future research should focus on large-scale, prospective validation studies to assess the real-world clinical impact of AI in AF management. Additionally, the integration of AI with multimodal data sources, such as genomics, imaging, and continuous physiological monitoring, holds potential for further enhancing personalized treatment strategies. With ongoing advancements, AI is poised to play an increasingly integral role in AF care, improving outcomes through more precise, data-driven, and individualized approaches.

## Figures and Tables

**Table 1:** Key studies investigating AI applications in AF catheter ablation optimization.

Study (Year)	Patient Population	Sample Size	AI Technique	Findings	Clinical Implications
Bahlke et al. (2024) [20]	Patients with long-standing persistent AF	50	Volta VX1 software for AI-guided ablation of spatio-temporal dispersions	82% of patients remained in stable sinus rhythm after an average of 1.46 procedures; 52% experienced arrhythmia recurrence; AF cycle length prolonged significantly; low complication rate.	AI-guided ablation of spatio-temporal dispersions may improve outcomes for persistent AF patients, but randomized trials are needed to confirm long-term efficacy.
Zou et al. (2024) [76]	Patients undergoing radiofrequency catheter ablation for AF	118	CARTOSOUND FAM AI-based ICE module for 3D LA reconstruction	98% acute ablation success rate; no immediate complications; mean procedure time: 136.9 min; mean RF time: 29.6 min; 92% received PVI, 68% received posterior wall isolation.	AI-integrated ICE module enables accurate LA reconstruction without a multipolar mapping catheter, demonstrating high acute success and safety; long-term AF recurrence needs further study.
Ogbomo-Harmitt et al. (2022) [77]	Persistent AF patients undergoing simulated RF catheter ablation	122	Deep learning (CNN) for predicting success of fibrosis-based and rotor-based ablation	For fibrosis-based ablation: AUC 0.92, recall 0.89, precision 0.82; for rotor-based ablation: AUC 0.77, recall 0.93, precision 0.76; saliency maps identified ablation lesions in 62–71% of cases.	DL-based prediction models can identify proarrhythmogenic regions and improve AI interpretability for AF ablation, potentially aiding clinical decision making.
Park et al. (2024) [78]	Patients undergoing de novo AF catheter ablation	5466	AI-estimated electrocardiographic age (AI-ECG) using ResNet-based model	AI-ECG age gap (≥10 years) associated with higher AF recurrence risk; 5-year recurrence HR 1.44 (95% CI 1.31–1.59); each year increase in AI-ECG age gap increased recurrence risk by 1%.	AI-ECG age gap is a potential predictor of AF recurrence after ablation, offering a simple and interpretable risk marker for clinical use.
TAILORED-AF Trial (2025) [17]	Patients with drug-refractory persistent or long-standing persistent AF	374	AI algorithm detecting spatio-temporal dispersion for tailored ablation	Freedom from AF at 12 months: 88% (tailored arm) vs. 70% (PVI-only arm); freedom from any arrhythmia: no significant difference after one procedure in the entire population, becoming significant after one or two procedures. Significant difference after a single procedure in the >6 months’ persistent AF pre-specified subgroup; tailored ablation had longer procedure and ablation times but similar safety outcomes.	AI-guided ablation targeting spatio-temporal dispersion improves AF elimination compared to PVI alone; long-term efficacy and need for additional AT ablation require further study.
Gruwez et al. (2024) [79]	Patients undergoing AF ablation with pre-procedure sinus rhythm ECG	53	Deep neural network (DNN)-based AI-enabled ECG algorithm	AI-ECG predicted AF recurrence with AUC 0.65; patients classified as high-risk had a 2.6-fold higher risk of AF recurrence (HR 2.6, *p* = 0.037).	AI-enabled ECG analysis may help predict AF recurrence risk post-ablation, potentially improving patient selection and risk stratification.
Fox et al. (2024) [80]	Patients undergoing AI-guided arrhythmia mapping for catheter ablation	28	Forward-solution AI ECG mapping system	Reduced time to first ablation by 19% (133 vs. 165 min, *p* = 0.02); reduced procedure duration by 22.6% (233 vs. 301 min, *p* < 0.001); reduced fluoroscopy time by 43.7% (18.7 vs. 33.2 min, *p* < 0.001); 6-month arrhythmia-free survival: 73.5% (AI) vs. 63.3% (control, *p* = 0.56).	AI-guided ECG mapping improves procedural efficiency by reducing mapping time, procedure duration, and radiation exposure without negatively affecting outcomes.
Asaeikheybari et al. (2024) [81]	Patients undergoing AF catheter ablation with pre-procedure CT scans	809	AI-based segmentation and radiomic analysis of pulmonary vein morphology	Primary PV morphology associated with AF recurrence (AUC 0.73, 0.71, 0.70 across datasets); AF+ cases exhibited greater surface complexity; secondary PV features had weaker association (AUC ~0.61).	AI-extracted pulmonary vein features may serve as predictors of AF recurrence post-ablation; potential for improved patient selection and ablation strategies.
Sato et al. (2024) [82]	Patients with persistent AF undergoing catheter ablation	497	Uplift modeling with adaptive boosting to predict benefit from extensive ablation	Uplift score ≥ 0.0124 identified patients who benefited from extensive ablation (HR 0.40, *p* = 0.015); no benefit observed in patients with uplift score < 0.0124 (HR 1.17, *p* = 0.661); creatinine, LVEF, BNP, hemoglobin among top predictors.	AI-based uplift modeling can stratify patients who require extensive catheter ablation, offering a precision medicine approach to AF ablation strategies.
Liu et al. (2020) [83]	Patients with paroxysmal AF undergoing catheter ablation	521	Deep learning (ResNet34) for predicting non-pulmonary vein (NPV) triggers	Prediction accuracy for NPV triggers: 88.6%; sensitivity: 75.0%, specificity: 95.7%; AUC for individual images: 0.82, for patients: 0.88.	AI-based prediction of NPV triggers before ablation may enhance procedural planning, helping to reduce AF recurrence and improve ablation strategies.

Abbreviations: AF—atrial fibrillation; AI—artificial intelligence; AUC—area under the curve; BNP—brain natriuretic peptide; CNN—convolutional neural network; CT—computed tomography; DNN—deep neural network; ECG—electrocardiogram; HR—hazard ratio; ICE—intracardiac echocardiography; LAA—left atrial appendage; LA—left atrium; LVEF—left ventricular ejection fraction; NPV—non-pulmonary vein; PVI—pulmonary vein isolation; PV—pulmonary vein; RF—radiofrequency; ResNet—residual neural network; SVC—superior vena cava.

## Data Availability

All data generated in this research are included within the article.

## References

[1] Tan S., Zhou J., Veang T., Lin Q., Liu Q. (2025). Global, Regional, and National Burden of Atrial Fibrillation and Atrial Flutter from 1990 to 2021: Sex Differences and Global Burden Projections to 2046-A Systematic Analysis of the Global Burden of Disease Study 2021. Europace.

[2] Kornej J., Börschel C.S., Benjamin E.J., Schnabel R.B. (2020). Epidemiology of Atrial Fibrillation in the 21st Century: Novel Methods and New Insights. Circ. Res..

[3] Linz D., Gawalko M., Betz K., Hendriks J.M., Lip G.Y.H., Vinter N., Guo Y., Johnsen S. (2024). Atrial Fibrillation: Epidemiology, Screening and Digital Health. Lancet Reg. Health Eur..

[4] Alonso A., Bengtson L.G.S. (2014). A Rising Tide: The Global Epidemic of Atrial Fibrillation. Circulation.

[5] Linz D., Andrade J.G., Arbelo E., Boriani G., Breithardt G., Camm A.J., Caso V., Nielsen J.C., De Melis M., De Potter T. (2024). Longer and Better Lives for Patients with Atrial Fibrillation: The 9th AFNET/EHRA Consensus Conference. Europace.

[6] Burdett P., Lip G.Y.H. (2022). Atrial Fibrillation in the UK: Predicting Costs of an Emerging Epidemic Recognizing and Forecasting the Cost Drivers of Atrial Fibrillation-Related Costs. Eur. Heart J. Qual. Care Clin. Outcomes.

[7] Esteva A., Chou K., Yeung S., Naik N., Madani A., Mottaghi A., Liu Y., Topol E., Dean J., Socher R. (2021). Deep Learning-Enabled Medical Computer Vision. NPJ Digit. Med..

[8] Lam T.Y.T., Cheung M.F.K., Munro Y.L., Lim K.M., Shung D., Sung J.J.Y. (2022). Randomized Controlled Trials of Artificial Intelligence in Clinical Practice: Systematic Review. J. Med. Internet Res..

[9] Plana D., Shung D.L., Grimshaw A.A., Saraf A., Sung J.J.Y., Kann B.H. (2022). Randomized Clinical Trials of Machine Learning Interventions in Health Care: A Systematic Review. JAMA Netw. Open.

[10] Zhang Y., Xu S., Xing W., Chen Q., Liu X., Pu Y., Xin F., Jiang H., Yin Z., Tao D. (2024). Robust Artificial Intelligence Tool for Atrial Fibrillation Diagnosis: Novel Development Approach Incorporating Both Atrial Electrograms and Surface ECG and Evaluation by Head-to-Head Comparison With Hospital-Based Physician ECG Readers. J. Am. Heart Assoc..

[11] Pamporis K., Karakasis P., Sagris M., Theofilis P., Milaras N., Pantelidaki A., Mourouzis I., Fragakis N., Vlachos K., Kordalis A. (2025). Prevalence of Asymptomatic Atrial Fibrillation and Risk Factors Associated with Asymptomatic Status: A Systematic Review and Meta-Analysis. Eur. J. Prev. Cardiol..

[12] Karakasis P., Pamporis K., Siontis K.C., Theofilis P., Samaras A., Patoulias D., Stachteas P., Karagiannidis E., Stavropoulos G., Tzikas A. (2024). Major Clinical Outcomes in Symptomatic vs. Asymptomatic Atrial Fibrillation: A Meta-Analysis. Eur. Heart J..

[13] Noseworthy P.A., Attia Z.I., Behnken E.M., Giblon R.E., Bews K.A., Liu S., Gosse T.A., Linn Z.D., Deng Y., Yin J. (2022). Artificial Intelligence-Guided Screening for Atrial Fibrillation Using Electrocardiogram during Sinus Rhythm: A Prospective Non-Randomised Interventional Trial. Lancet.

[14] Shajari S., Kuruvinashetti K., Komeili A., Sundararaj U. (2023). The Emergence of AI-Based Wearable Sensors for Digital Health Technology: A Review. Sensors.

[15] Jung S., Song M.-K., Lee E., Bae S., Kim Y.-Y., Lee D., Lee M.J., Yoo S. (2022). Predicting Ischemic Stroke in Patients with Atrial Fibrillation Using Machine Learning. Front. Biosci..

[16] Labovitz D.L., Shafner L., Reyes Gil M., Virmani D., Hanina A. (2017). Using Artificial Intelligence to Reduce the Risk of Nonadherence in Patients on Anticoagulation Therapy. Stroke.

[17] Deisenhofer I., Albenque J.-P., Busch S., Gitenay E., Mountantonakis S.E., Roux A., Horvilleur J., Bakouboula B., Oza S., Abbey S. (2025). Artificial Intelligence for Individualized Treatment of Persistent Atrial Fibrillation: A Randomized Controlled Trial. Nat. Med..

[18] Seitz J., Mohr Durdez T., Lotteau S., Bars C., Pisapia A., Gitenay E., Monteau J., Reist M., Serdi M., Dayot A. (2024). Artificial Intelligence-Adjudicated Spatiotemporal Dispersion: A Patient-Unique Fingerprint of Persistent Atrial Fibrillation. Heart Rhythm.

[19] Seitz J., Durdez T.M., Albenque J.P., Pisapia A., Gitenay E., Durand C., Monteau J., Moubarak G., Théodore G., Lepillier A. (2022). Artificial Intelligence Software Standardizes Electrogram-Based Ablation Outcome for Persistent Atrial Fibrillation. J. Cardiovasc. Electrophysiol..

[20] Bahlke F., Englert F., Popa M., Bourier F., Reents T., Lennerz C., Kraft H., Martinez A.T., Kottmaier M., Syväri J. (2024). First Clinical Data on Artificial Intelligence-Guided Catheter Ablation in Long-Standing Persistent Atrial Fibrillation. J. Cardiovasc. Electrophysiol..

[21] Attia Z.I., Noseworthy P.A., Lopez-Jimenez F., Asirvatham S.J., Deshmukh A.J., Gersh B.J., Carter R.E., Yao X., Rabinstein A.A., Erickson B.J. (2019). An Artificial Intelligence-Enabled ECG Algorithm for the Identification of Patients with Atrial Fibrillation during Sinus Rhythm: A Retrospective Analysis of Outcome Prediction. Lancet.

[22] Sehrawat O., Kashou A.H., Noseworthy P.A. (2022). Artificial Intelligence and Atrial Fibrillation. J. Cardiovasc. Electrophysiol..

[23] Christopoulos G., Graff-Radford J., Lopez C.L., Yao X., Attia Z.I., Rabinstein A.A., Petersen R.C., Knopman D.S., Mielke M.M., Kremers W. (2020). Artificial Intelligence-Electrocardiography to Predict Incident Atrial Fibrillation: A Population-Based Study. Circ. Arrhythm. Electrophysiol..

[24] Khurshid S., Friedman S., Reeder C., Di Achille P., Diamant N., Singh P., Harrington L.X., Wang X., Al-Alusi M.A., Sarma G. (2022). ECG-Based Deep Learning and Clinical Risk Factors to Predict Atrial Fibrillation. Circulation.

[25] Wu C., Hwang M., Huang T.-H., Chen Y.-M.J., Chang Y.-J., Ho T.-H., Huang J., Hwang K.-S., Ho W.-H. (2021). Application of Artificial Intelligence Ensemble Learning Model in Early Prediction of Atrial Fibrillation. BMC Bioinform..

[26] Rabinstein A.A., Yost M.D., Faust L., Kashou A.H., Latif O.S., Graff-Radford J., Attia I.Z., Yao X., Noseworthy P.A., Friedman P.A. (2021). Artificial Intelligence-Enabled ECG to Identify Silent Atrial Fibrillation in Embolic Stroke of Unknown Source. J. Stroke Cerebrovasc. Dis..

[27] Yao X., Attia Z.I., Behnken E.M., Walvatne K., Giblon R.E., Liu S., Siontis K.C., Gersh B.J., Graff-Radford J., Rabinstein A.A. (2021). Batch Enrollment for an Artificial Intelligence-Guided Intervention to Lower Neurologic Events in Patients with Undiagnosed Atrial Fibrillation: Rationale and Design of a Digital Clinical Trial. Am. Heart J..

[28] Rosman L., Lampert R., Zhuo S., Li Q., Varma N., Burg M., Gaffey A.E., Armbruster T., Gehi A. (2024). Wearable Devices, Health Care Use, and Psychological Well-Being in Patients With Atrial Fibrillation. J. Am. Heart Assoc..

[29] Manetas-Stavrakakis N., Sotiropoulou I.M., Paraskevas T., Maneta Stavrakaki S., Bampatsias D., Xanthopoulos A., Papageorgiou N., Briasoulis A. (2023). Accuracy of Artificial Intelligence-Based Technologies for the Diagnosis of Atrial Fibrillation: A Systematic Review and Meta-Analysis. J. Clin. Med..

[30] Sattar Y., Song D., Sarvepalli D., Zaidi S.R., Ullah W., Arshad J., Mir T., Zghouzi M., Elgendy I.Y., Qureshi W. (2022). Accuracy of Pulsatile Photoplethysmography Applications or Handheld Devices vs. 12-Lead ECG for Atrial Fibrillation Screening: A Systematic Review and Meta-Analysis. J. Interv. Card. Electrophysiol..

[31] Pereira T., Tran N., Gadhoumi K., Pelter M.M., Do D.H., Lee R.J., Colorado R., Meisel K., Hu X. (2020). Photoplethysmography Based Atrial Fibrillation Detection: A Review. NPJ Digit. Med..

[32] Kitajima H., Takeda K., Ishizawa M., Aihara K., Minamino T. (2025). Detection of Atrial Fibrillation from Pulse Waves Using Convolution Neural Networks and Recurrence-Based Plots. Chaos.

[33] Perez M.V., Mahaffey K.W., Hedlin H., Rumsfeld J.S., Garcia A., Ferris T., Balasubramanian V., Russo A.M., Rajmane A., Cheung L. (2019). Large-Scale Assessment of a Smartwatch to Identify Atrial Fibrillation. N. Engl. J. Med..

[34] Guo Y., Wang H., Zhang H., Liu T., Liang Z., Xia Y., Yan L., Xing Y., Shi H., Li S. (2019). Mobile Photoplethysmographic Technology to Detect Atrial Fibrillation. J. Am. Coll. Cardiol..

[35] Mannhart D., Lischer M., Knecht S., du Fay de Lavallaz J., Strebel I., Serban T., Vögeli D., Schaer B., Osswald S., Mueller C. (2023). Clinical Validation of 5 Direct-to-Consumer Wearable Smart Devices to Detect Atrial Fibrillation: BASEL Wearable Study. JACC. Clin. Electrophysiol..

[36] Fallet S., Lemay M., Renevey P., Leupi C., Pruvot E., Vesin J.-M. (2019). Can One Detect Atrial Fibrillation Using a Wrist-Type Photoplethysmographic Device?. Med. Biol. Eng. Comput..

[37] Schack T., Safi Harb Y., Muma M., Zoubir A.M. Computationally Efficient Algorithm for Photoplethysmography-Based Atrial Fibrillation Detection Using Smartphones. Proceedings of the 2017 39th Annual International Conference of the IEEE Engineering in Medicine and Biology Society (EMBC).

[38] Turakhia M.P., Desai M., Hedlin H., Rajmane A., Talati N., Ferris T., Desai S., Nag D., Patel M., Kowey P. (2019). Rationale and Design of a Large-Scale, App-Based Study to Identify Cardiac Arrhythmias Using a Smartwatch: The Apple Heart Study. Am. Heart J..

[39] Fine J., Branan K.L., Rodriguez A.J., Boonya-Ananta T., Ajmal, Ramella-Roman J.C., McShane M.J., Coté G.L. (2021). Sources of Inaccuracy in Photoplethysmography for Continuous Cardiovascular Monitoring. Biosensors.

[40] Rizas K.D., Freyer L., Sappler N., von Stülpnagel L., Spielbichler P., Krasniqi A., Schreinlechner M., Wenner F.N., Theurl F., Behroz A. (2022). Smartphone-Based Screening for Atrial Fibrillation: A Pragmatic Randomized Clinical Trial. Nat. Med..

[41] Rodrigo M., Alhusseini M.I., Rogers A.J., Krittanawong C., Thakur S., Feng R., Ganesan P., Narayan S.M. (2022). Atrial Fibrillation Signatures on Intracardiac Electrograms Identified by Deep Learning. Comput. Biol. Med..

[42] Li Y.-G., Pastori D., Farcomeni A., Yang P.-S., Jang E., Joung B., Wang Y.-T., Guo Y.-T., Lip G.Y.H. (2019). A Simple Clinical Risk Score (C(2)HEST) for Predicting Incident Atrial Fibrillation in Asian Subjects: Derivation in 471,446 Chinese Subjects, With Internal Validation and External Application in 451,199 Korean Subjects. Chest.

[43] Alonso A., Krijthe B.P., Aspelund T., Stepas K.A., Pencina M.J., Moser C.B., Sinner M.F., Sotoodehnia N., Fontes J.D., Janssens A.C.J.W. (2013). Simple Risk Model Predicts Incidence of Atrial Fibrillation in a Racially and Geographically Diverse Population: The CHARGE-AF Consortium. J. Am. Heart Assoc..

[44] Lip G.Y.H., Nieuwlaat R., Pisters R., Lane D.A., Crijns H.J.G.M. (2010). Refining Clinical Risk Stratification for Predicting Stroke and Thromboembolism in Atrial Fibrillation Using a Novel Risk Factor-Based Approach: The Euro Heart Survey on Atrial Fibrillation. Chest.

[45] Chen D., Liu S., Kingsbury P., Sohn S., Storlie C.B., Habermann E.B., Naessens J.M., Larson D.W., Liu H. (2019). Deep Learning and Alternative Learning Strategies for Retrospective Real-World Clinical Data. NPJ Digit. Med..

[46] Tiwari P., Colborn K.L., Smith D.E., Xing F., Ghosh D., Rosenberg M.A. (2020). Assessment of a Machine Learning Model Applied to Harmonized Electronic Health Record Data for the Prediction of Incident Atrial Fibrillation. JAMA Netw. Open.

[47] Suenari K., Chao T.-F., Liu C.-J., Kihara Y., Chen T.-J., Chen S.-A. (2017). Usefulness of HATCH Score in the Prediction of New-Onset Atrial Fibrillation for Asians. Medicine.

[48] Chamberlain A.M., Agarwal S.K., Folsom A.R., Soliman E.Z., Chambless L.E., Crow R., Ambrose M., Alonso A. (2011). A Clinical Risk Score for Atrial Fibrillation in a Biracial Prospective Cohort (from the Atherosclerosis Risk in Communities [ARIC] Study). Am. J. Cardiol..

[49] Schnabel R.B., Sullivan L.M., Levy D., Pencina M.J., Massaro J.M., D’Agostino R.B.S., Newton-Cheh C., Yamamoto J.F., Magnani J.W., Tadros T.M. (2009). Development of a Risk Score for Atrial Fibrillation (Framingham Heart Study): A Community-Based Cohort Study. Lancet.

[50] Hill N.R., Ayoubkhani D., McEwan P., Sugrue D.M., Farooqui U., Lister S., Lumley M., Bakhai A., Cohen A.T., O’Neill M. (2019). Predicting Atrial Fibrillation in Primary Care Using Machine Learning. PLoS ONE.

[51] Sekelj S., Sandler B., Johnston E., Pollock K.G., Hill N.R., Gordon J., Tsang C., Khan S., Ng F.S., Farooqui U. (2021). Detecting Undiagnosed Atrial Fibrillation in UK Primary Care: Validation of a Machine Learning Prediction Algorithm in a Retrospective Cohort Study. Eur. J. Prev. Cardiol..

[52] Tseng A.S., Noseworthy P.A. (2021). Prediction of Atrial Fibrillation Using Machine Learning: A Review. Front. Physiol..

[53] Nadarajah R., Wu J., Frangi A.F., Hogg D., Cowan C., Gale C. (2021). Predicting Patient-Level New-Onset Atrial Fibrillation from Population-Based Nationwide Electronic Health Records: Protocol of FIND-AF for Developing a Precision Medicine Prediction Model Using Artificial Intelligence. BMJ Open.

[54] Nadarajah R., Wahab A., Reynolds C., Raveendra K., Askham D., Dawson R., Keene J., Shanghavi S., Lip G.Y.H., Hogg D. (2023). Future Innovations in Novel Detection for Atrial Fibrillation (FIND-AF): Pilot Study of an Electronic Health Record Machine Learning Algorithm-Guided Intervention to Identify Undiagnosed Atrial Fibrillation. Open Heart.

[55] Li H., Gao M., Song H., Wu X., Li G., Cui Y., Li Y., Xie Z., Ren Q., Zhang H. (2023). Predicting Ischemic Stroke Risk from Atrial Fibrillation Based on Multi-Spectral Fundus Images Using Deep Learning. Front. Cardiovasc. Med..

[56] Seners P., Turc G., Oppenheim C., Baron J.-C. (2015). Incidence, Causes and Predictors of Neurological Deterioration Occurring within 24 h Following Acute Ischaemic Stroke: A Systematic Review with Pathophysiological Implications. J. Neurol. Neurosurg. Psychiatry.

[57] Siegler J.E., Martin-Schild S. (2011). Early Neurological Deterioration (END) after Stroke: The END Depends on the Definition. Int. J. Stroke.

[58] Kim S.-H., Jeon E.-T., Yu S., Oh K., Kim C.K., Song T.-J., Kim Y.-J., Heo S.H., Park K.-Y., Kim J.-M. (2021). Interpretable Machine Learning for Early Neurological Deterioration Prediction in Atrial Fibrillation-Related Stroke. Sci. Rep..

[59] Vinter N., Frederiksen A.S., Albertsen A.E., Lip G.Y.H., Fenger-Grøn M., Trinquart L., Frost L., Møller D.S. (2020). Role for Machine Learning in Sex-Specific Prediction of Successful Electrical Cardioversion in Atrial Fibrillation?. Open Heart.

[60] Emren S.V., Kocabaş U., Duygu H., Levent F., Şimşek E.Ç., Yapan Emren Z., Tülüce S. (2016). The Role of HATCH Score in Predicting the Success Rate of Sinus Rhythm Following Electrical Cardioversion of Atrial Fibrillation. Kardiol. Pol..

[61] Vitali F., Serenelli M., Airaksinen J., Pavasini R., Tomaszuk-Kazberuk A., Mlodawska E., Jaakkola S., Balla C., Falsetti L., Tarquinio N. (2019). CHA2DS2-VASc Score Predicts Atrial Fibrillation Recurrence after Cardioversion: Systematic Review and Individual Patient Pooled Meta-Analysis. Clin. Cardiol..

[62] Nuñez-Garcia J.C., Sánchez-Puente A., Sampedro-Gómez J., Vicente-Palacios V., Jiménez-Navarro M., Oterino-Manzanas A., Jiménez-Candil J., Dorado-Diaz P.I., Sánchez P.L. (2022). Outcome Analysis in Elective Electrical Cardioversion of Atrial Fibrillation Patients: Development and Validation of a Machine Learning Prognostic Model. J. Clin. Med..

[63] Lee G., Baker E., Collins R., Merino J.L., Desteghe L., Heidbuchel H. (2022). The Challenge of Managing Multimorbid Atrial Fibrillation: A Pan-European European Heart Rhythm Association (EHRA) Member Survey of Current Management Practices and Clinical Priorities. Europace.

[64] Guerra J.M., Moreno Weidmann Z., Perrotta L., Sultan A., Anic A., Metzner A., Providencia R., Boveda S., Chun J. (2024). Current Management of Atrial Fibrillation in Routine Practice According to the Last ESC Guidelines: An EHRA Physician Survey-How Are We Dealing with Controversial Approaches?. Europace.

[65] Karakasis P., Patoulias D., Popovic D.S., Pamporis K., Theofilis P., Nasoufidou A., Stachteas P., Samaras A., Tzikas A., Giannakoulas G. (2024). Effects of Mineralocorticoid Receptor Antagonists on New-Onset or Recurrent Atrial Fibrillation: A Bayesian and Frequentist Network Meta-Analysis of Randomized Trials. Curr. Probl. Cardiol..

[66] Stachteas P., Nasoufidou A., Karagiannidis E., Patoulias D., Karakasis P., Alexiou S., Samaras A., Zormpas G., Stavropoulos G., Tsalikakis D. (2024). The Role of Sodium Glucose Co-Transporter 2 Inhibitors in Atrial Fibrillation: A Comprehensive Review. J. Clin. Med..

[67] Karakasis P., Fragakis N., Patoulias D., Theofilis P., Kassimis G., Karamitsos T., El-Tanani M., Rizzo M. (2024). Effects of Glucagon-Like Peptide 1 Receptor Agonists on Atrial Fibrillation Recurrence After Catheter Ablation: A Systematic Review and Meta-Analysis. Adv. Ther..

[68] Allen M.J., Nichols D.J., Oliver S.D. (2000). The Pharmacokinetics and Pharmacodynamics of Oral Dofetilide after Twice Daily and Three Times Daily Dosing. Br. J. Clin. Pharmacol..

[69] Sedgwick M.L., Rasmussen H.S., Cobbe S.M. (1992). Effects of the Class III Antiarrhythmic Drug Dofetilide on Ventricular Monophasic Action Potential Duration and QT Interval Dispersion in Stable Angina Pectoris. Am. J. Cardiol..

[70] Attia Z.I., Sugrue A., Asirvatham S.J., Ackerman M.J., Kapa S., Friedman P.A., Noseworthy P.A. (2018). Noninvasive Assessment of Dofetilide Plasma Concentration Using a Deep Learning (Neural Network) Analysis of the Surface Electrocardiogram: A Proof of Concept Study. PLoS ONE.

[71] Lee H., Kim H.J., Chang H.W., Kim D.J., Mo J., Kim J.-E. (2021). Development of a System to Support Warfarin Dose Decisions Using Deep Neural Networks. Sci. Rep..

[72] Chen C., Yin C., Wang Y., Zeng J., Wang S., Bao Y., Xu Y., Liu T., Fan J., Liu X. (2023). XGBoost-Based Machine Learning Test Improves the Accuracy of Hemorrhage Prediction among Geriatric Patients with Long-Term Administration of Rivaroxaban. BMC Geriatr..

[73] Yao X., Abraham N.S., Alexander G.C., Crown W., Montori V.M., Sangaralingham L.R., Gersh B.J., Shah N.D., Noseworthy P.A. (2016). Effect of Adherence to Oral Anticoagulants on Risk of Stroke and Major Bleeding Among Patients With Atrial Fibrillation. J. Am. Heart Assoc..

[74] Zhang Z., Wang Z., Wang X., Wang K., Yuan Y., Li Q. (2024). A Novel Network with Enhanced Edge Information for Left Atrium Segmentation from LGE-MRI. Front. Physiol..

[75] Karakasis P., Theofilis P., Vlachakis P.K., Korantzopoulos P., Patoulias D., Antoniadis A.P., Fragakis N. (2024). Atrial Fibrosis in Atrial Fibrillation: Mechanistic Insights, Diagnostic Challenges, and Emerging Therapeutic Targets. Int. J. Mol. Sci..

[76] Zou F., Ammirati G., Ventrella N., Aguilera J., Marazzato J., Freilich M., Serna J.C., Lin A.N., La Fazia V.M., Mohanty S. (2024). PO-03-147 Atrial Fibrillation Ablation Using A 3D Artificial Intelligence Module Integration With Intracardiac Echocardiography. Heart Rhythm..

[77] Ogbomo-Harmitt S., Muffoletto M., Zeidan A., Qureshi A., King A., Aslanidi O. (2022). Can Artificial Intelligence Prediction of Successful Atrial Fibrillation Catheter Ablation Therapy Be Interpretable?. Eur. Heart J. Digit. Health.

[78] Park H., Kwon O.-S., Shim J., Kim D., Park J.-W., Kim Y.-G., Yu H.T., Kim T.-H., Uhm J.-S., Choi J.-I. (2024). Artificial Intelligence Estimated Electrocardiographic Age as a Recurrence Predictor after Atrial Fibrillation Catheter Ablation. NPJ Digit. Med..

[79] Gruwez H., Barthels M., Dhont S., Meekers E., Wouters F., Pierlet N., Nuyens D., Rivero-Ayerza M., Van Herendael H., Pison L. (2024). Predicting Atrial Fibrillation Recurrence after Catheter Ablation Using an Artificial Intelligence-Enabled Electrocardiogram Algorithm. EP Eur..

[80] Fox S.R., Toomu A., Gu K., Kang J., Sung K., Han F.T., Hoffmayer K.S., Hsu J.C., Raissi F., Feld G.K. (2024). Impact of Artificial Intelligence Arrhythmia Mapping on Time to First Ablation, Procedure Duration, and Fluoroscopy Use. J. Cardiovasc. Electrophysiol..

[81] Asaeikheybari G., El-Harasis M., Gupta A., Shoemaker M.B., Barnard J., Hunter J., Passman R.S., Sun H., Kim H.S., Schilling T. (2024). Artificial Intelligence-Based Feature Analysis of Pulmonary Vein Morphology on Computed Tomography Scans and Risk of Atrial Fibrillation Recurrence After Catheter Ablation: A Multi-Site Study. Circ. Arrhythm. Electrophysiol..

[82] Sato T., Sotomi Y., Hikoso S., Kitamura T., Nakatani D., Okada K., Dohi T., Sunaga A., Kida H., Matsuoka Y. (2024). Uplift Modeling to Identify Patients Who Require Extensive Catheter Ablation Procedures among Patients with Persistent Atrial Fibrillation. Sci. Rep..

[83] Liu C.-M., Chang S.-L., Chen H.-H., Chen W.-S., Lin Y.-J., Lo L.-W., Hu Y.-F., Chung F.-P., Chao T.-F., Tuan T.-C. (2020). The Clinical Application of the Deep Learning Technique for Predicting Trigger Origins in Patients With Paroxysmal Atrial Fibrillation With Catheter Ablation. Circ. Arrhythm. Electrophysiol..

[84] Chen H.-H., Liu C.-M., Chang S.-L., Chang P.Y.-C., Chen W.-S., Pan Y.-M., Fang S.-T., Zhan S.-Q., Chuang C.-M., Lin Y.-J. (2020). Automated Extraction of Left Atrial Volumes from Two-Dimensional Computer Tomography Images Using a Deep Learning Technique. Int. J. Cardiol..

[85] Liu X., Shen Y., Zhang S., Zhao X. (2018). Segmentation of Left Atrium Through Combination of Deep Convolutional and Recurrent Neural Networks. J. Med. Imaging Health Inform..

[86] Muffoletto M., Fu X., Roy A., Varela M., Bates P.A., Aslanidi O.V. Development of a Deep Learning Method to Predict Optimal Ablation Patterns for Atrial Fibrillation. Proceedings of the 2019 IEEE Conference on Computational Intelligence in Bioinformatics and Computational Biology (CIBCB).

[87] Brooks S., Metzner A., Wohlmuth P., Lin T., Wissner E., Tilz R., Rillig A., Mathew S., Saguner A., Heeger C. (2018). Insights into Ablation of Persistent Atrial Fibrillation: Lessons from 6-Year Clinical Outcomes. J. Cardiovasc. Electrophysiol..

[88] Chiang C.-E., Naditch-Brûlé L., Murin J., Goethals M., Inoue H., O’Neill J., Silva-Cardoso J., Zharinov O., Gamra H., Alam S. (2012). Distribution and Risk Profile of Paroxysmal, Persistent, and Permanent Atrial Fibrillation in Routine Clinical Practice: Insight from the Real-Life Global Survey Evaluating Patients with Atrial Fibrillation International Registry. Circ. Arrhythm. Electrophysiol..

[89] Valderrábano M., Peterson L.E., Swarup V., Schurmann P.A., Makkar A., Doshi R.N., DeLurgio D., Athill C.A., Ellenbogen K.A., Natale A. (2020). Effect of Catheter Ablation With Vein of Marshall Ethanol Infusion vs Catheter Ablation Alone on Persistent Atrial Fibrillation: The VENUS Randomized Clinical Trial. JAMA.

[90] Marrouche N.F., Wazni O., McGann C., Greene T., Dean J.M., Dagher L., Kholmovski E., Mansour M., Marchlinski F., Wilber D. (2022). Effect of MRI-Guided Fibrosis Ablation vs Conventional Catheter Ablation on Atrial Arrhythmia Recurrence in Patients With Persistent Atrial Fibrillation: The DECAAF II Randomized Clinical Trial. JAMA.

[91] Kistler P.M., Chieng D., Sugumar H., Ling L.-H., Segan L., Azzopardi S., Al-Kaisey A., Parameswaran R., Anderson R.D., Hawson J. (2023). Effect of Catheter Ablation Using Pulmonary Vein Isolation With vs Without Posterior Left Atrial Wall Isolation on Atrial Arrhythmia Recurrence in Patients With Persistent Atrial Fibrillation: The CAPLA Randomized Clinical Trial. JAMA.

[92] Lebert J., Ravi N., Fenton F.H., Christoph J. (2021). Rotor Localization and Phase Mapping of Cardiac Excitation Waves Using Deep Neural Networks. Front. Physiol..

[93] Liao S., Ragot D., Nayyar S., Suszko A., Zhang Z., Wang B., Chauhan V.S. (2021). Deep Learning Classification of Unipolar Electrograms in Human Atrial Fibrillation: Application in Focal Source Mapping. Front. Physiol..

[94] Tang S., Razeghi O., Kapoor R., Alhusseini M.I., Fazal M., Rogers A.J., Rodrigo Bort M., Clopton P., Wang P.J., Rubin D.L. (2022). Machine Learning-Enabled Multimodal Fusion of Intra-Atrial and Body Surface Signals in Prediction of Atrial Fibrillation Ablation Outcomes. Circ. Arrhythm. Electrophysiol..

[95] Seitz J., Bars C., Théodore G., Beurtheret S., Lellouche N., Bremondy M., Ferracci A., Faure J., Penaranda G., Yamazaki M. (2017). AF Ablation Guided by Spatiotemporal Electrogram Dispersion Without Pulmonary Vein Isolation: A Wholly Patient-Tailored Approach. J. Am. Coll. Cardiol..

